# Question order in the assessment of misperception of physical activity

**DOI:** 10.1186/1479-5868-4-42

**Published:** 2007-09-20

**Authors:** Catherine Bolman, Lilian Lechner, Marius van Dijke

**Affiliations:** 1 Faculty of Psychology, Open University of the Netherlands, PO Box 2960, 6401 DL Heerlen, The Netherlands

## Abstract

**Background:**

People often have misperceptions (overestimation or underestimation) about the health-related behaviours they engage in, which may have adverse consequences for their susceptibility to behavioural change. Misperception is usually measured by combining and comparing quantified behavioural self-reports with subjective classification of the behaviour. Researchers assume that such assessments of misperception are not influenced by the order of the two types of measurement, but this has never been studied. Based on the precaution adoption model and the information processing theory, it might be expected that taking the subjective measurement after a detailed quantified behavioural self-report would improve the accuracy of the subjective measurement because the quantified report urges a person to think more in detail about their own behaviour.

**Methods:**

In an experiment (n = 521), quantified self-report and subjective assessment were manipulated in a questionnaire. In one version, the quantified self-report was presented before the subjective assessment, whereas in the other version, the subjective assessment came first.

**Results:**

Neither subjective assessment nor overestimation of physical activity were biased by the order of the questions. Underestimation was more prevalent among subgroups of the group which answered the subjective assessment after the quantified self-report.

**Conclusion:**

Question order in questionnaires does not seem to influence misperceptions concerning physical activity in groups relevant for health education (overestimators: those who do not meet the guidelines for  physical activity while rating their physical activity as sufficient or  high). The small order effect found in underestimators is less relevant for health education because this subgroup already meets the guideline and therefore does not need to change behaviour.

## Background

Studies show that people often have misperceptions about the health-related behaviours they engage in [1-12]. People usually assume that their behaviour is adequate to prevent disease even if in reality it does not meet the health guidelines. Studies have shown that people often overestimate their physical activity level [5-7] and their fruit and vegetable consumption [8-10], but underestimate their fat intake [11] and alcohol consumption [4]. Misperceptions often relate to behaviours for which there is no obvious dividing line between what is healthy or unhealthy or adequate to prevent disease [12]. For example, whereas it is well-known that smoking is bad for someone's health, and people obviously know whether they smoke or not, it is less clear what exactly constitutes a healthy diet and when a persons' diet is healthy or adequate to prevent disease.

Misperceptions may have adverse consequences for people's susceptibility to behavioural change and health education. According to the Precaution Adoption Process Model [13,14], people need to be aware of their own risk behaviour before they are susceptible to behavioural change and may change their risk behaviour. Furthermore, it is likely that people with misperceptions about their own behaviour do not pay attention to messages in health education interventions because they think these messages do not apply to them. Studies of the consequences of misperception have shown that people who think that their behaviour is adequate have less intention to change [4-6,15]. In terms of their attitude, self-efficacy and the social influence they perceive, these people are comparable to those who actually behave in accordance with the health guidelines [4-6,15].

A few studies have assessed the determinants of misperception [4,12,16]. These studies, on fruit [4,16], vegetable [4,16], alcohol consumption [4] and physical activity [12], showed that misperception is correlated with the way in which and the extent to which persons make interpersonal comparisons. People who erroneously classify themselves as behaving in a healthy way are more likely to compare themselves with those who are perceived to engage in equally healthy or less healthy behaviour (downward comparison) and rate their own behaviour as healthier than that of these others (optimistic bias) [12,16].

Misperceptions about health-related behaviour are often measured by combining and comparing two types of self-report [6,12,15,17,18]. The first type is a self-report in which the behaviour is assessed in detail by quantitative measurements, often by means of validated questionnaires [19-21]. The behaviour is subsequently scored on the basis of accepted guidelines for health-enhancing behaviours. Instruments frequently used for such quantified behavioural self-reports are the validated Short Questionnaire to Assess Health Enhancing physical activity (SQUASH) [19] and the validated 25-item fat intake food frequency questionnaire [20,21]. The scores are then compared with the relevant guidelines to determine whether people do or do not meet these health guidelines. The second type of self-report provides a subjective (self-rated) general evaluative estimation of the behaviour [4,6,17,18]. Self-rated dietary fat intake, for example, is measured by asking respondents to evaluate their fat intake on a bipolar five-point scale including answering categories ranging from very low fat intake to very high fat intake [17]. Misperception is then assessed by comparing the subjective self-rated estimation of behaviour with the findings of the quantified behavioural self-report indicating whether subjects meet the health guideline.

A fair amount of research has been done into the degree of misperceptions about health-related behaviours and their causes and consequences in terms of behavioural change. In addition, recently developed interventions incorporate strategies to make people aware of their risk behaviour [10,18]. To our knowledge, however, it has so far never been studied whether the order in which the quantified behavioural self-report and subjective assessment are presented in questionnaires can influence a person's judgement of the behaviour and therefore the degree of misperception, while studies have varied in the order in which the quantified behavioural self-report and subjective assessment are presented in the questionnaires. No standardized order instruction seems to exist. This suggests that researchers have always assumed that the order in which questions are presented does not affect the measurement of misperception by means of questionnaires. If this assumption is correct, we can continue to measure misperception by comparing subjective and quantified behavioural self-reports, with the order of the questions making no difference.

Previous studies in the area of question-order effects, however, have demonstrated systematic differences in people's responses that could be attributed to the order in which questions were presented [22-24]. This might imply that question order could also influence the extent of misperception. Therefore, this study assessed whether the order of the subjective and quantified behavioural self-reports influenced the results (i.e. the extent of misperception). Answering this question is relevant to future studies that try to explain and change health behaviours in general and those on misperceptions about health behaviours in particular. We chose physical activity behaviour as the behaviour to test this on.

The main question this study tried to answer was thus whether the order of presentation of the questions asking for subjective and quantified behavioural self-reports on physical activity influences the subjective self-report and the corresponding degree of misperception. We therefore manipulated the order of the questions in a questionnaire.

As mentioned above, it would be good for the evaluation of findings of past and future research into health promotion practices if we found that the order of the two types of question did not influence the measurement of misperceptions. This would justify the measurement procedure used so far. Nevertheless, the studies on question-order effects referred to above suggested that there might be an influence. Furthermore, based on the Precaution Adoption Process Model [13,14] and the Information Processing Theory [25], we considered it likely that filling in a detailed quantified questionnaire on physical activity behaviour would help respondents become aware of their own behaviour (in terms of risk) and would therefore result in a more accurate subjective estimation of the behaviour (i.e. in less misperception). Based on these two theories [13,14,25], we therefore expected that a detailed quantified behavioural self-report in our study would activate prior knowledge (revealing respondents' own activities and knowledge about physical activity behaviour) and would induce respondents to think about their own behaviour in more detail. This was then expected to result in fewer misperceptions (in terms of overestimation and underestimation) about the behaviour.

In addition, we explored the effects of people's feeling of involvement in physical activity and their reasons to be physically active (e.g., health, weight control) on the occurrence of misperception and their possible relationships with the order of questions. With respect to the perceived feeling of involvement, the Elaboration Likelihood Model suggests that persons with high issue involvement process information rational by means of a central route of processing, and therefore estimate their behaviour more accurately than those who show low involvement in the issue [26]. When involvement is low, people tend to follow the 'peripheral route' of processing, which is characterized by less cognitive effort and more reliance on simple situational cues. We expected that if a detailed quantified behavioural self-report was administered first, people might be more likely to switch to a more central and cognitive processing of information, resulting in greater awareness of their own behaviour and therefore less misperception.

With respect to reasons to be physically active, prior research has shown that people have other reasons besides health promotion for being active (also known as outcome expectancies, e.g. loss of weight or relaxation) [12,27-29]. Lechner and colleagues suggested that people use these reasons as a reference point to rate their behaviour in terms of adequacy, instead of or in addition to the health reference point that health educators use [12]. They found that people's perceptions or misperceptions about their own behaviour were related to their reasons for being physically active. They found, for example, that people who overestimated their physical activity behaviour had higher scores on 'feeling fit' as a reason for them to be active than those who estimated their physical activity to be low. Since involvement and reasons to be active might be expected to influence people's perceptions of the sufficiency of their behaviour, we took these factors into account in our study of the effect of question order on misperception. We explored the role of these factors not only as main predictors of misperception but also as possible moderators in the relationship between question order and misperception. A reason to take these possible moderators into account is that a recent study showed that question-order effects in assessing the perceived importance of skin cancer were moderated by the extent to which people were involved with the issue [24]. Theoretically based it might be expected that strongly involved persons are less susceptible for item order since they use a more accurate and systematic way of processing information compared to low involved persons [26].

In sum, the main question of this study was whether the order of presentation of the questions asking for subjective assessment and quantified behavioural self-report on physical activity influences the subjective self-report and the corresponding degree of misperception. In addition, we explored the effects of people's feeling of involvement in physical activity and their reasons to be physically active (e.g., health, weight control) on the occurrence of misperception and their possible relationships with the order of questions.

## Methods

### Design

The study used an experimental design in which a quantified behavioural self-report and a subjective assessment of behaviour were manipulated in a questionnaire. Two versions of the questionnaire were used, one for each experimental condition. In one version, the quantified behavioural self-report part of the questionnaire was presented before the subjective assessment part (condition QS), whereas in the other version, the subjective assessment was presented first, followed by the quantified behavioural self-report (condition SQ). In all other respects, the two versions of the questionnaire were identical.

### Procedure

The questionnaires were pretested among a random sample of persons from the general Dutch population (N = 12). The actual study sample was then derived from data administered by a national telephone guide organization. Only persons who had given the organization permission to disclose their address for research purposes could be included. Questionnaires (N = 1000) were sent to participants' home addresses together with a letter explaining the goal of the study ('to study physical activity among the Dutch population'). They could return the questionnaire anonymously, in a prepaid envelope. A postal reminder was sent to all participants two weeks after the first mailing.

### Questionnaire

As mentioned, the two questionnaires differed in the order in which the part asking for a subjective assessment and the part requiring a quantified behavioural self-report were presented. In all other aspects, the questionnaires were identical. They started with questions on demographics (gender, age, educational level and complaints restricting physical activity), followed by the parts with the subjective assessment and the quantified behavioural self-report, whose order was varied. The final part of the questionnaire consisted of questions on feelings of involvement and reasons to be physically active.

The subjective assessment used the question "What do you think of the amount of physical activity you engage in?" (bipolar 5-point answering scale, ranging from very low (1) to sufficient (3) to very high (5) [5,6]. The quantified behavioural self-report used the SQUASH instrument [19,30], which had been validated in a previous study with the help of a Computer Science and Applications (CSA) inc. activity monitor [19]. The correlation coefficient for validity in that study was 0.45 (95% CI 0.17–0.66). The exact agreement between the SQUASH scores and those of the CSA was 46% and the weighted kappa 0.30. Correlations for the reproducibility of the individual questions had a mean value of 0.75 and varied between 0.44 and 0.96 [19]. SQUASH is widely accepted as an instrument to measure physical activity and has been used in a number of studies [e.g. [30-32]]. It is considered an acceptable substitute for objective activity measures like accelerometers, heart rate counters and other observation instruments [19,30]. SQUASH [33] consists of 14 questions on a total of four domains of physical activity (i.e. commuting activities like walking to/from work; leisure time activities like gardening and sports; household activities like cooking and activities at work and/or school, like regularly lifting heavy objects at work). The questions assess the number of days per week on which respondents engage in an activity, the average time spent on that activity per day and the intensity of the activity. Based on these questions, an overall SQUASH score on physical activity is calculated using an algorithm corrected for age, because age determines the intensity of an activity.

The overall SQUASH score was then compared with the Dutch guidelines for healthy physical activity (NNGB guideline) [34] and the international guidelines of the American College of Sports Medicine [35], resulting in two groups of people. Persons were meeting the physical activity guidelines if they were at least moderately physically active for a minimum of five days a week for at least 30 minutes a day (received score 1). All others were categorized as not meeting the physical activity guidelines (score 0).

**Figure 1:**
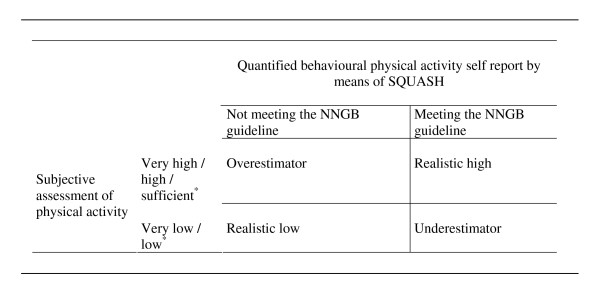
Classification of respondents in categories of misperception^6^. * answering categories in subjective assessment.

To assess misperception, the dichotomised 'do or do not meet the guideline' score was compared with the scores from the subjective assessment. For this purpose, the scores on the subjective assessment were dichotomised into the categories 'very low or low physical activity in the respondent's opinion' (score 0) and 'sufficient or high physical activity in the respondent's opinion' (score 1). Respondents were subsequently allocated to one of the four categories shown in Figure 1. The *Realistic high group *consisted of participants who met the physical activity guideline and rated their physical activity as sufficient or high. *Overestimators *were those who did not meet the physical activity guideline and rated their physical activity as sufficient or high. *Underestimators *were those who met the physical activity guideline though rating their physical activity as low or very low. The *Realistic low group *included those who did not meet the physical activity guideline and rated their physical activity as low or very low. Overestimators and underestimators could be labelled the 'misperception group', while the realistic high and realistic low participants had no misperception.

For the purpose of the additional research question, the questionnaire included questions on feelings of involvement and reasons to be physically active. Feelings of involvement were assessed by two questions that were averaged (r = .64). One question was "How important is it for you to be physically active?" (bipolar 5-point answering scale ranging from very important (1) to very unimportant (5)). Reasons to be physically active were assessed by means of six outcome expectancies. Respondents were asked to what extent six potential outcomes of physical activity indicated in the questions (health, weight control, stress reduction, appearance, feeling fit, relaxation) were important reasons for them to be sufficiently physically active. The chosen outcome expectations were based on a previous study of De Ridder and Lechner [5]. Respondents had to mark the importance they attached to each reason on a 4-point unipolar scale (no reason (score 1) to very important reason (score 4)). These questions were regarded as separate items.

### Analyses

Demographic differences between the two experimental conditions were tested using chi-square statistics for the categorical and dichotomous variables (gender, educational level, complaints restricting physical activity, meeting the NNGB guideline) and a t-test for age.

Pearson correlations between the total score on physical activity (based on SQUASH) and the score on the subjective assessment were computed for the whole group and for the QS and SQ groups separately. Four t-tests were conducted. One t-test was used to assess whether those who did meet the NNGB guideline differed in their subjective estimation of physical activity from those who did not meet this guideline. Two t-tests were conducted to assess whether the SQ and QS groups differed in their subjective estimation of physical activity and in the quantified behavioural self-report scores. In addition, Z-transformed mean scores of the difference between the objective and subjective measurements of behaviour were calculated for the QS and SQ groups separately and tested by means of a t-test.

To find out whether the order of the two assessments influenced the degree of misperception (main research question), a linear regression analysis was conducted with subjective assessment as the dependent variable and the order of the two assessments as the independent factor, corrected for quantified behavioural self-report (total SQUASH score).

Next, chi-square tests were used to examine whether respondents in the two conditions differed in the observed frequencies of the 4 categories (i.e., high realistics respondents, overestimators, underestimators, low realistics, Figure 1).

Two types of analyses were used to answer the additional research question, i.e. the effect of a person's involvement and reasons to be physically active on the occurrence of misperception and their possible relationship with the order of the questions. Two-way Anova's with Games-Howell post-hoc analyses [36] were conducted for the four categories of Figure 1, while hierarchical logistic regression analyses were carried out in two separate groups [one group in which overestimators were compared with realists (i.e., the combination of realistic low and realistic high categories) and one group in which underestimators were compared with realists. These analyses in separate groups were needed since overestimators and underestimators had to be compared separately with realists to prevent reciprocal influences (if overestimation and underestimation were taken together, their scores would average out). In the first logistic regression analysis, underestimators were omitted and overestimators were compared with realists. In the second logistic regression analysis, overestimators were omitted and underestimators were compared with realists. In both analyses, the main effects of the order of the questions, involvement and reasons to be active were tested by entering them as one block in the first step. Interactions were added in step two, using a hierarchical backward elimination procedure. Non-significant interaction terms (P < .10) were removed one by one [37]. If any significant interaction remained between question order and one of the other variables, the effect of order was stratified to this variable.

Demographic variables were not controlled for in the hierarchical logistic regression analyses nor in the linear regression analyses, since the experimental groups did not differ in demographic characteristics. All analyses were done using SPSS 11.0.5 for Windows [38].

## Results

### Demographic characteristics and differences in conditions

A total of 521 persons returned the questionnaire (52% response). Five respondents were excluded due to a large number of missing values in their questionnaires (>50%), so questionnaires of 516 responders were used.

The sample included 44% men and 55% women, while 1% had not indicated their sex. The mean age was 53.7 years (SD = 17.8; range = 19–91). Thirty-one percent had followed primary education, junior general secondary education or preparatory secondary vocational education, 31% had followed senior general secondary education, senior secondary vocational education or pre-university education, and 38% had followed higher professional education or university. Almost 70% indicated not to be restricted in physical activity by (physical) complaints. Physical activity was found to be important to the respondents (M = 1.9; SD = 0.7; range 1 (very important) to 5 (very unimportant)). They also felt involved with being physically active (M = 2.2; SD = 0.8; range: 1 (very involved) to 5 (very uninvolved). Almost 17% of the participants were in the realistic low category, 16% were overestimators, 15% underestimators and 52% were in the realistic high category. This means that 67% of the respondents met the NNGB guideline and that misperception (overestimation plus underestimation) was prevalent in 31% of the respondents. Of those who did not meet the guideline, 48% estimated their physical activity to be sufficient or high.

The QS group consisted of 246 respondents, the SQ group of 270. Experimental groups did not differ in demographic characteristics or in meeting the NNGB guideline.

### Relation between quantified behavioural self-report and subjective assessment and effect of question order

An *r *of .41 was found for the total score on physical activity and the subjective assessment, while individual correlations for the QS and SQ groups were .38 and .43. All p-values were < .01. A t-test showed that those who did not meet the norm of the NNGB guideline had significantly lower scores for subjective estimation of their physical activity level (M = 2.44, SD = .87) than those who did meet the guideline (M = 2.98, SD = .79), (t(512) = -6.78, P < .0001). Z-transformed mean scores for the difference between the quantified behavioural self-report and subjective assessments of behaviour were not significantly different between the QS (M = .056, SD = 1.09) and SQ (M = -.047, SD = 1.10) groups (t(512) = 1.075, P = .28). T-tests showed no differences between the QS and SQ groups in terms of the total SQUASH scores nor in the scores on the subjective assessment of activity. Means of the total SQUASH scores for the QS and SQ groups were 7.55 (SD = 5.35) and 7.49 (5.38), respectively (t(514) = 0.135, P = .89). Means of the subjective assessment scores of the QS and SQ groups were 2.76 (SD = 0.81) and 2.84 (0.88), respectively (t(512) = -0.994, P = .32). The linear regression analyses showed that quantified behavioural self-report explained 41% of the variance in subjective assessment (t(1, 513) = 10.04, β = .41, P < .0001) while question order did not add any significant explanation (β = .05, P = .24).

Table 1 shows how misperception was distributed over the two experimental conditions, which did not differ significantly. Misperception (underestimators plus overestimators) was prevalent in 33.6% of the QS group compared to 28.9% in the SQ group.

**Table 1:** Misperception categories in the QS en SQ conditions (%)*

Condition	Realistic low category	Overestimators	Underestimators	Realistic high category
QS	16	16.4	17.2	50.4
SQ	17.8	15.2	13.7	53.3

*X^2 ^= 1.6; df = 3; P = .66

### Role of involvement and reasons to be active in misperception

The Anova analyses (Table 2) showed that the four categories differed significantly in their mean scores on involvement and on all reasons to be physically active, except for weight control. The two experimental conditions (QS versus SQ) only resulted in different scores for 'being physically active for relaxation', though at p = .06. A post hoc t-test revealed however no significant differences between the two conditions (QS group (M = 2.54, SD = 0.98) and SQ group (M = 2.40, SD = 0.98), (t(481) = 1.569, P = .12). Feelings of involvement and all other reasons to be active did not differ between the QS and SQ groups. Nor were interaction effects found between question order and feeling of involvement or reasons to be active.

**Table 2:** Summary of results of Anova analyses of differences between the four categories in terms of involvement and reasons to be active, in relation to the order of the questions

Condition	Main effect: F (df), p-value	Interaction effect: F (df), p-value
Health	F (3, 505) = 4.47, p < .01	F (3, 505) = .27, p = .85
Question order	F (3, 505) = .98, p = .32	
		
Weight	F (3, 489) = .74, p = .53	F (3, 489) = .56, p = .64
Question order	F (3, 489) = 1.16, p = .28	
		
Stress reduction	F (3, 482) = 4.26, p < .01	F (3, 482) = .91, p = .44
Question order	F (3, 482) = .52, p = .47	
		
Appearance	F (3, 477) = 3.70, p < .05	F (3, 477) = .187, p = .13
Question order	F (3, 477) = .01, p = .92	
		
Feeling fit	F (3, 495) = 2.97, p < .05	F (3, 495) = .55, p = .46
Question order	F (3, 495) = 1.60, p = .19	
		
Relaxation	F (3, 482) = 7.30, p < .0001	F (3, 482) = .68, p = .57
Question order	F (3, 482) = 3.52, p = .06	
		
Involvement	F (3, 506) = 22.45, p < .0001	F (3, 506) = 1.12, p = .34
Question order	F (3, 506) = .001, p = .97	

Table 3 shows, for each of the four groups, the results of the post-hoc analyses of scores on involvement and reasons to be physically active. The greatest differences were found between the realistic high and realistic low groups. Realistic high estimators considered health, appearance, relaxation and stress relief more important reasons to be physically active than realistic low estimators. Overestimators mentioned feeling fit and relaxation as more important reasons to be physically active than realistic low estimators and underestimators. Both groups, realistic high and overestimators, expressed a stronger sense of involvement in physical activity than the participants who estimated their physical activity to be low (underestimators and realistic low).

**Table 3:** Differences in reasons to active and involvement between the groups realistic high, underestimation, overestimation, and realistic low, using two-way Anova^a^

	Realistic high (Rh)	Overestimation (Ov)	Underestimation (Un)	Realistic low (Rl)	Differences p < .05
Health	3.42	3.38	3.21	3.16	Rh>Rl
Appearance	2.47	2.40	2.30	2.08	Rh>Rl
Feeling fit	3.33	3.27	3.07	3.04	Rh>Un, Rl
Weight	2.84	2.72	2.87	2.70	n.s.
Relaxation	2.60	2.63	2.20	2.15	Rh, Ov>Un, Rl
Stress relief	2.46	2.40	2.14	2.00	Rh>Rl
Involvement^b^	1.83	2.04	2.30	2.40	Rh, Ov>Un, Rl

Answering scores ranging from 1 (no reason) to 4 (very important reason); ^a^Analyses included main effects of reasons to be active, involvement and order of the questions and their interaction effects, for post-hoc analyses Games-Howell procedure was used because of the unequal group sizes as recommended by Field [36]; ^b^Scale score ranging from 1(very important) to 5 (very unimportant).

Hierarchical logistic regression analysis using underestimation (scored as 1) versus no misperception (scored as 0 (reference group) as the dependent variable and order, involvement and the six reasons to be active as predictors showed a main effect of involvement, a borderline significant effect of question order and interactions between (1) health as a reason to be active and order and (2) involvement and order (Table 4). Since interactions may cause unreliable odds ratios and p-values [37], four additional logistic regression analyses were carried out in which the effects of order were tested for a number of subgroups, based on 'health as a reason to be active' and 'involvement'. For the purpose of these stratified analyses, two subgroups were formed on the basis of 'health as a reason to be active'. One subgroup consisted of respondents who stated that health was an important or very important reason for them to be active (n = 380), while the other subgroup consisted of people for whom health was no reason or only a minor reason to be active (n = 55). We also formed a subgroup with respondents who were not or hardly involved in physical activity (n = 67) and a group of involved or highly involved respondents (n = 366). Table 5 shows the results of the stratified analyses for each of the four subgroups. Among those who considered health to be an important or very important reason to be physically active, the SQ condition resulted in less underestimation, though at borderline significance. In those who were not involved in physical activity, the SQ condition also resulted in less underestimation, also at borderline significance. No effects of question order were found in the other subgroups.

**Table 4:** Summary of logistic regression predicting underestimation behaviour (n = 397)^a,b^

	β coefficient	SE	Odds Ratio	P^c^
*Step one*				
Question order^d^	-.46	.27	0.63	.09
Involvement^e^	.60	.22	1.83	.005
*Step two*				
Question order	4.54	2.11	93	.03
Involvement	1.09	.33	2.97	.001
Health	.42	.36	1.52	.25
Order with involvement (interaction)	-.90	.43	0.41	.04
Order with health (interaction)	-.93	.46	0.39	.04

^a^Demographic characteristics were not controlled for since groups did not differ in these characteristics; ^b^Only significant variables are reported; ^c^Wald test statistic; ^d^QS is reference category; ^e^Higher score means lower involvement.

**Table 5:** Summary of stratified analyses on the effects of question order on underestimation^a^

	β coefficient	SE	Odds Ratio	P^b^
Those who found health an *important *reason to be active (n = 380)				
Question order^c^	-.54	.27	0.63	.09
Those who found health an *unimportant *reason to be active (n = 55)				
Question order^c^	.87	.97	2.39	.32
Those who are *involved *with being active (n = 366)				
Question order^c^	-.16	.29	0.85	.58
Those who are *not involved *with being active (n = 67)				
Question order^c^	-.87	.53	0.42	.10

^a^Demographic characteristics were not controlled for since groups did not differ in these characteristics; ^b^Wald test statistic; ^c^QS is the reference category

**Figure 2:**
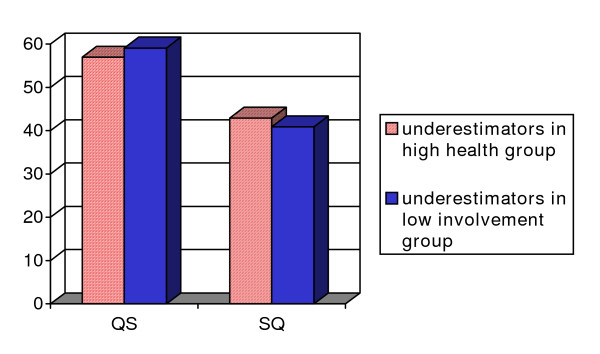
Percentages of underestimators among subgroups^a^. ^a^borderline significant differences (p < .10) in percentages.

In line with the results of the regression analyses, chi-square analyses showed lower percentages of underestimators in the SQ group among those who mentioned health as an important reason to be active (X^2 ^(1, 380) = 2.94, p = .08) and among those who were not involved in physical activity (X^2^(1, 67) = 2.71, p = .10) (see Figure 2 for percentages). These differences were however only borderline significant.

Hierarchical logistic regression analysis with overestimation (1) versus no misperception (0) as the dependent variable and order, involvement and the six reasons to be physically active as the predictors showed no main effects and no interactions.

## Discussion

The aim of this study was to find out whether the order of questions relating to a quantified behavioural self-report and a subjective assessment of physical activity behaviour influences the assessment of misperception in this behaviour. This question is relevant to studies on physical activity, health behaviours in general and to studies on misperception in the area of health education and health promotion.

In line with other studies [4,5,15], we found a low correlation between the quantified behavioural self-report and the subjective assessment of behaviour, a correlation which hardly differed between the two conditions (quantified self-report followed by subjective assessment (QS) or the other way round (SQ)). The results of the t-tests and linear regression showed no effect of the order in which the questions were presented on the subjective estimation of behaviour. Our Anova analysis of misperception from the perspective of four categories (underestimators, overestimators, realistic high estimators, realistic low estimators, all relative to the Dutch national guideline, Figure 1) did not show an influence of question order on the occurrence of misperception either. Nor did it reveal any interaction effects between outcome expectations, involvement and question order. The Anova analyses did, however, show that the four categories differed from each other in terms of involvement and of most outcome expectations. People who perceived their physical activity level as adequate (realistic high estimators and overestimators) had higher scores on all reasons to be active (except weight) than those who did not perceive their activity behaviour as adequate (realistic low estimators and underestimators). People with misperceptions did not differ in their judgment on the importance of health from those without misperception. There were two exceptions: overestimators saw relaxation as a more important reason to be active and expressed less involvement in physical activity than realistic low estimators and underestimators. The findings suggest that people may use not only health but also other benchmarks (e.g. relaxation) in their subjective estimation of the adequacy of their behaviour. The results do not, however, suggest that only misperceptors (i.e. overestimators and underestimators) use different benchmarks for assessing their physical activity involvement. For a detailed analysis of the differences between the categories in terms of involvement and outcome expectancies, we refer to a previous study by Lechner, Bolman and Van Dijke [12].

A comparison between overestimators (with respect to physical activity behaviour the most important category for health education and behavioural change) and realists in the logistic regression analysis did not find any effect of question order either.

Our comparison of underestimators with realists showed that involvement in physical activity predicted underestimation of the behaviour, while the effect of the order of questions was borderline significant. Underestimators of physical activity are, however, not very interesting to health educators, since these persons are already meeting the national Dutch (NNGB) guideline and therefore do not need to change their physical activity behaviour. This means that there is no urgency for health educators to invest in this group. In order to increase public health, they should primarily invest in persons who are not meeting the guideline (overestimators and low realists). From the perspective of the aim of this study, the important finding is that the extent of overestimation is not influenced by the order of the questions. Involvement and health moderated the effect of question order on the degree of underestimation. Stratified analyses among those who perceived health as an important or very important reason to be physically active showed that those who filled in the quantified behavioural self report before the subjective assessment were more likely to underestimate their physical activity than those who completed them in the opposite order. The same was true for those who were less involved in physical activity. Both findings were borderline significant. Rimal and Real [24], who assessed the perceived importance of skin cancer related to issue involvement and question-order effects, also found an interaction effect between involvement and question order. Like us, these researchers found that high involvement was not associated with question-order effects, but low involvement was (at borderline significance in our study). This is in agreement with the Elaboration Likelihood Model, which assumes greater context-related effects (i.e. those of question order) among respondents with low involvement, because they process information via a peripheral route [26]. It is harder to understand why those who rate health as the most important reason to be active are affected by question order (at borderline significance in our study).

Contrary to what was expected from the Precaution Adoption model [13,14] and the Information Processing theory [25], the quantified behavioural self-report did not seem to help respondents who did not meet the NNGB guideline to accurately estimate their own behaviour. This suggests that more is needed to estimate one's behaviour appropriately. Recent studies showed the efficacy and effectiveness of interventions that incorporate detailed quantified behavioural self-reports as a diagnostic tool, in combination with feedback, on correct estimation of fruit, vegetable and fat intake [18,39]. This implies that correcting misperceptions always requires a combination of detailed quantified behavioural self-report and feedback. The feedback should include information on whether the person meets the guideline.

Taken together, the results show for the categories that are of interest to health educators, i.e. overestimators and realistic low estimators, that assessing misperception by comparing quantified behavioural self-report and subjective self report is valid and reliable. The order of the quantified behavioural self-report and the subjective assessment questions makes no difference and does not influence the subjective assessment. This is an important result for researchers in the area of health behaviours and misperception of health behaviours.

The present study showed that 67% of the respondents met the NNGB guideline, while 16% overestimated and 15% underestimated their physical activity. Overestimators were those who subjectively estimated their behaviour as sufficient while not actually meeting the NNGB guideline, as derived from the quantified behavioural self-report assessment by means of the SQUASH instrument. Underestimators were those who did meet the guideline, though they subjectively estimated their behaviour as insufficient. Half of those who did not meet the NNGB guideline thought that they were doing fine (the so-called overestimators). We found 10% less misperception than Ronda and colleagues [6], though the percentage was comparable to that found by De Ridder and Lechner [5]. This might be partly explained by the fact that those researchers used a different instrument for the quantified self-report of physical activity. Our quantified assessment of physical activity with SQUASH [19] was more detailed, and corrected for age. Also in line with the study by De Ridder & Lechner, but different from other studies [6,15,40,41], was our finding that two thirds of the respondents met the NNGB norm; the other studies reported 40–55%. Again, these differences might be related to the instruments that were used, and the age correction in the SQUASH. In agreement with the guidelines proposed by Wendel-Vos & Schuit [33], the algorithm we used in the present study to calculate the total SQUASH score took age into account in calculating intensity categories (light, moderate and vigorous) for physical activities. For example, vigorous activities for 55+ participants meant ≥ 5 MET, while for adults they were ≥ 6.5 MET. Previous studies may not have applied the age corrections.

Our study was limited by the fact that our sample did not reflect the general Dutch population. Our sample was slightly older and more likely to meet the guideline for physical activity [40,41]. This may have had consequences for the reported physical activity and the possible predictors of misperception, and may therefore affect the generalizability of the results. Also, our response rate, though comparable to that in most other similar studies, was only slightly over 50%. It is, however, unlikely that these aspects have affected the results of the experiment (i.e., the effect of the order of quantified behavioural self-report and subjective assessment questions). The nature of our research question reduced the need for generalizability. The study may have suffered from type I errors due to multiple testing. Although we used a validated and widely accepted quantified behavioural self-report scale of physical activity (SQUASH), we could have strengthened our study by collecting corroborating data that would support the validity of this instrument (e.g., accelerometers). We only studied the effect of question order on misperception in self-administered questionnaires, and it is possible that question order effects on misperception might exist in telephone-administered interviews, another important mode of data collection in health education research. This is suggested by several experimental studies that showed clear question-order effects in telephone surveys, while not finding such effects in self-administered questionnaires [42,43].

Future research should examine the cognitive process underlying the subjective estimation of behaviour by respondents, as well as the phenomenon of optimistic bias. It would also be useful to study whether the theoretical assumption of our study, i.e. that detailed quantified behavioural self-reports activate prior knowledge of the behaviour, is true and whether the degree of misperception decreases if the subjective assessment is more detailed and includes the guideline. A question related to this issue is whether the rather broad question used for the subjective assessment sufficiently captured aspects like habitual physical activity. It is also interesting to know what time period participants have in mind when they are asked to give a subjective estimation of their behaviour. Another question that needs further study is whether our findings can be generalized to other behaviours (e.g., dietary behaviour or alcohol consumption).

## Conclusion

For those who wish to use combined quantified behavioural self-reports and subjective assessments of physical activity for research purposes or intervention development, our findings allow the conclusion that the procedure used so far in research to assess misperception in physical activity is valid and reliable. In assessing the prevalence of overestimation in physical activity behaviour, they do not need to be concerned about the order of the questions in their assessment.
